# Neonatal outcomes in pregnancies resulting from oocyte donation: a cohort study in Sweden

**DOI:** 10.1186/s12887-016-0708-5

**Published:** 2016-10-21

**Authors:** Evangelia Elenis, Gunilla Sydsjö, Alkistis Skalkidou, Claudia Lampic, Agneta Skoog Svanberg

**Affiliations:** 1 Department of Women’s and Children’s Health, Uppsala University, Uppsala University Hospital, SE-751 83 Uppsala, Sweden; 2 Obstetrics and Gynecology, Department of Clinical and Exprerimental Medicine, Faculty of Health Sciences, Linköping University, Linköping, Sweden; 3 Department of Neurobiology, Care sciences and Society, Karolinska Institutet, Solna, Sweden

**Keywords:** Donor conception, Indication for oocyte donation, IVF, Neonatal morbidity, Neonatal outcomes, Newborn, Oocyte donation

## Abstract

**Background:**

Pregnancies resulting through oocyte donation have been associated with increased risk for adverse outcomes for the mother, such as gestational hypertensive disorders. However, little is known about possible neonatal complications of such pregnancies. The purpose of this study was to evaluate the neonatal health outcomes among singleton pregnancies in a population of relatively young and healthy oocyte recipients in Sweden, taking into account the medical indication leading to treatment.

**Methods:**

This cohort study involved 76 women conceiving with donated oocytes, 149 age-matched nulliparous women conceiving spontaneously and 63 women conceiving after non-donor IVF. Participants were recruited during 2005–2008 and followed up until delivery. Data on neonatal outcomes were retrieved from the National Birth Medical Register and the medical records of oocyte recipients from seven Swedish University Hospitals with IVF clinics. Logistic regression analyses were performed to examine the association of mode of conception and neonatal outcomes, adjusted for maternal age and BMI, gestational age and delivery by cesarean section.

**Results:**

Infants conceived through oocyte donation had higher odds for premature delivery [OR 2.36, 95 % CI (1.02–5.45)], for being small for gestational age [OR 4.23, 95 % CI (1.03–17.42)] and having Apgar score below 7 at 5 min [OR 10.57, 95 % CI (1.21–92.20)] compared to spontaneously conceived infants. Similar trends were observed when comparing infants conceived through oocyte donation to those conceived by traditional IVF. Furthermore, donor oocyte infants had a lower mean birthweight and length compared to autologous oocyte neonates (*p* = 0.013); however no differences were noted among infants born at term. Neonatal outcomes were more favorable among women with diminished ovarian reserve compared to those with other indications for oocyte donation.

**Conclusions:**

Infants conceived after oocyte donation in Sweden have higher odds of being born prematurely and having lower mean birthweight in comparison to non-donor infants.

It seems that these unfavorable neonatal outcomes are present despite the age, weight and health restrictions applied to recipients before oocyte donation treatment in Sweden.

## Background

Oocyte donation is a well established-widely applied infertility treatment for women with premature idiopathic, iatrogenic and even natural menopause, with more than 30.437 cycles per year being performed in Europe during 2012 [1]. Previous studies have reported an association between oocyte-donation pregnancies and an increased occurrence of hypertensive disorders of pregnancy [2–9], gestational diabetes [10], placental abnormalities [9, 11, 12], preterm delivery [2, 4, 11–13], prolonged maternal hospitalization after delivery and increased prevalence of caesarean section [4, 7, 13].

Regarding neonatal complications, the outcomes seem to be overall reassuring especially among singleton deliveries. The prevalence of major congenital malformations [6, 10] as well as the Apgar score [2, 4, 6] were noted to be comparable to that in the general population. However conflicting findings have been reported regarding neonatal hospital stay [2, 14, 15], birthweight [2, 4, 5, 9, 15, 16], rate of low birthweight (i.e. birthweight <2500 gr) [4, 5, 9, 17] and small for gestational age (SGA) infants [5, 15, 16]. In fact, in the recent meta-analysis by Adams et al. [18] the significant findings include being born with low birthweight (<2500 g), very low birthweight (<1500 g), preterm (<37^th^ week), with lower gestational age and preterm with low birthweight when compared with autologous oocyte counterparts. Some of the unfavorable outcomes have previously been attributed mostly to advanced maternal age, as well as the presence of multifetal pregnancies and prematurity as a consequence [13, 18].

Prompted by the observations mentioned above, we aimed to examine if medically indicated oocyte donation is associated with increased risk for adverse neonatal outcomes in singleton pregnancies in a population of relatively young and healthy oocyte recipients in Sweden and whether this is affected by treatment indication.

## Methods

### Study sample and data collection

The present observational cohort study is a sequel to the study evaluating the obstetric outcomes in pregnancies resulting from oocyte donation by Elenis et al. [7] and is part of the larger cohort study ”Swedish multicenter study on gamete donation” [19]. During the period 2005–2008, consecutive couples starting concurrently IVF treatment with either donated or autologous oocytes at University Hospitals in Stockholm, Gothenburg, Uppsala, Umeå, Linköping, Örebro and Malmö (Sweden) were approached regarding participation. In particular, women undergoing IVF with donated oocytes (*n* = 238) *vs* women treated with autologous oocytes (*n* = 233) were originally approached, a proportion of which (63 % *vs* 51 % respectively) were included in the larger cohort. Only women who became pregnant with autologous or donated oocytes and delivered singletons during the study period were included in this sequel study. All participating women were enrolled just once in the study and no repeat pregnancies were included.

The Index group (*n* = 76) is composed of women receiving treatment with donated oocytes who later gave birth to a singleton. In order to assess differences in outcome, two comparison groups were used;

- Comparison group A (CgA) (*n* = 149) comprises nulliparous women with spontaneously conceived pregnancies, singleton deliveries and no history of subfertility found in the medical register. Women in Comparison group A were matched to the Index group in regard to age in three categories, ≤29, 30–34, ≥35 years, at a ratio of 2:1. With the exception of the eligibility criteria according to the study design, Comparison group A was otherwise randomly selected.
- Comparison group B(CgB) (*n* = 63) comprises women undergoing IVF treatment with their own gametes due to couple infertility who conceived with singleton pregnancies at the University hospitals mentioned above. Women undergoing IVF with partner’s sperm were included as a Comparison group in order to assess if the adverse outcomes for oocyte recipients reported formerly can be credited solely to donation or perhaps even to the characteristics of the infertile couple, the underlying infertility and/or the use of assisted reproductive techniques [20]. Age matching was not performed.

All participants in Index and Comparison group B could communicate adequately in Swedish [19] and informed consent was obtained after written and oral information was given to the participants. No personal information on participants of Comparison group A was received and thus personal informed consent was not required for that group. Due to study design (i.e. being a sequel of the larger multicenter study on gamete donation), as well as the lack of appropriate and robust publications on the field before conducting this study, no power calculation had been performed a priori. All medical data analyzed were retrieved from the Swedish Medical Birth Register (MBR), which is a validated register including information on prenatal, delivery and neonatal care [21, 22]. Additional medical information for the oocyte recipients was extracted from the treatment protocols that are part of the medical records at each center.

The estimation of gestational age at delivery for women conceiving spontaneously was calculated mainly based on second-trimester ultrasound scan (which is attended by 98 % of pregnant women during 16^th^-19^th^ gestational week) [23] or on last menstrual period (if ultrasound scan was unavailable). For women subjected to conventional IVF and oocyte donation treatment, gestational age was estimated based on the date of the embryo transfer. All medical outcomes studied were retrieved from the MBR and the diagnosis according to the 10th version of the International Classification of Diseases (ICD-10) that the woman or infant had received perinatally (i.e. through pregnancy and up to 28 days after delivery). The set of neonatal variables explored was selected based on prior Swedish register studies (MBR) and other international studies on the field [18, 21]. No information was retrieved from the medical records of the neonates. It should be added that the rate of Small for Gestational Age (SGA) (i.e. weight for gestational age corresponding to more than two standard deviations below the Swedish growth standard) [24] was calculated either through ultrasound measurements antenatally or by comparing the expected weight to the actual birthweight postnatally.

### Medical background characteristics

Maternal background characteristics are presented with detailed demographic and clinical data in the preceding publication by Elenis et al. [7]. Briefly, the study groups did not differ significantly regarding the health status of the participants (data not shown); the only exception being hypothyroidism (7 oocyte recipients *vs* 1 person in Comparison group A and 0 in Comparison group B), nearly half of which could be credited to Turner syndrome, probably due to meticulous preconceptional health control of Turner women in Sweden [25]. The chronic medical conditions reported were otherwise previously described in detail by Elenis et al. [7]. Physicians at the Swedish university fertility clinics are practically unanimous in taking the position that women with serious intercurrent diseases should be denied gamete donation treatment; thus, only women with ascertained health status were included in the Index group. Regarding parity, the entire Comparison group A were nulliparous, in contrast to 92.1 % of participants in Comparison group B and 92.1 % of women in the Index group (data not shown) [7]. Additionally, oocyte recipients were diagnosed more frequently with hypertensive disorders of pregnancy compared to naturally conceiving women (15.8 % versus 6 %; *p* = 0.017) (data not shown) [7]. A summary of the most important baseline characteristics of the index and comparison groups is included even in this publication (Table 1).

**Table 1:** Maternal baseline characteristics between Index group (oocyte donation group) and Comparison group A (women having conceived spontaneously) and B (women having conceived through IVF)

	Index group		Comparison group A (CgA)		Comparison group B (CgB)		*p*-value Index vs CgA	*p*-value Index vs CgB
	n	%	n	%	n	%		
Maternal age (yrs)								
<35	36/76	47.4	81/149	54.4	42/63	66.7	*p* = 0.321	*p* = 0.002
≥35	40/76	52.6	68/149	45.6	21/63	33.3		
Maternal BMI(kg/m^2^)								
<25	33/70	47.1	87/132	65.9	40/62	64.5	*p* = 0.010	*p* = 0.045
≥25	37/70	52.9	45/132	34.1	22/62	35.5		
Caesarean Section								
No	34/76	44.7	110/149	73.8	51/63	81.0	*p* = 0.000	*p* = 0.000
Yes	42/76	55.3	39/149	26.2	12/63	19.0		

Our study does not include any incidents of maternal death. However, one case of in utero stillbirth in the 29^th^ week of gestation in Comparison group A was observed (excluded in the current sequel substudy) [7]. Although the original cohort is the same, the calculations differ since one participant was excluded due to stillbirth as mentioned above.

Finally, since previous reports have found a negative association between diminished ovarian reserve and birthweight [26], we chose to include in our study the medical indication leading to oocyte donation [7]. The most common reason for receiving donated oocytes in our study group was premature ovarian insufficiency (POI) or being “poor responder” defined according to the Bologna criteria [27] (37/76, 48.7 %), followed by Turner syndrome (10/76, 13.2 %); bilateral oophorectomy or post chemotherapy (9/76, 11.8 %); ”egg factor” (6/76, 7.9 %); multiple unsuccessful IVF cycles (5/76, 6.6 %) or genetic reasons (5/76, 6.6 %). Only 4 persons could not be classified according to the above categories (5.3 %). For more details please see the publication by Elenis et al. [7]. It should be added that “egg factor” is a generally poorly defined category associated with sufficient quantity but somehow defective quality of oocytes resulting in infertility.

### Statistical analysis

Data analysis was conducted using IBM SPSS v.20 (IBM Inc., Armonk, NY, USA). Statistical significance was set at a *p*-value of <0.05 (two-sided) in all analyses.

The association between various neonatal outcomes and Index/Comparison group status was evaluated by first comparing Index women (oocyte recipients) to Comparison group A (spontaneously pregnant women) and then to Comparison group B (women having conceived with conventional IVF). Normality of the data was evaluated using the Shapiro-Wilk test. The groups were compared with the use of Mann Whitney U test (for continuous variables non-normally distributed), Chi square or Fisher’s exact test (for categorical variables) as well as logistic regression analyses; a single regression model without adjustment for socio-demographic factors, as well as a multivariate logistic regression model. Covariates likely to affect the outcomes were chosen based on results from prior studies and are included in the multivariate logistic regression model: maternal age as completed years on delivery day (two categories, <35 years or ≥35 years); body mass index (BMI, kg/m^2^) defined as BMI recorded at first antenatal visit (two categories, <25 kg/m^2^ or ≥ 25 kg/m^2^) [28]; gestational age at delivery as a continuous variable and delivery by caesarean section (no/yes). When studying outcomes such as SGA & LGA diagnosis and preterm delivery, the multivariate regression model was not adjusted for gestational length, since by definition the variables were already corrected for gestational age. After employing a path diagram (such as *DAGitty* graphic model) preeclampsia was considered as a mediator between mode of conception and neonatal outcomes and was therefore not selected as a confounder to be included in the statistical models. Women who conceived spontaneously or by conventional IVF (Comparison group A and B respectively) were chosen as reference groups in the logistic regression models. The results were expressed as odds ratios (OR) and the corresponding 95 % confidence intervals (CI) were estimated.

Finally, a within-group analysis regarding the most frequent neonatal outcomes and Index/Control status was performed by group comparison with Chi-square or Fisher’s exact test (for categorical variables) or Mann Whitney U test (for numerical variables non-normally distributed). More specifically, the Index group after being divided in two subgroups including women with “diminished ovarian reserve” or “other indication” for OD treatment was compared in turn to Comparison group A with regard to various neonatal outcomes.

## Results

The maternal baseline characteristics of the Index and Control women are presented in Table 1. Oocyte recipients have a more advanced age compared to Comparison group B, are more often overweight or obese and deliver more often by cesarean section compared to both Comparison groups (Table 1).

Neonatal outcomes are presented in Tables 2 and 3 where infants of oocyte recipients are compared to infants who were conceived spontaneously and after conventional IVF, respectively. Regarding perinatal mortality, one neonatal death associated to chromosome deletion occurred within the Index group; the infant was delivered in the 34^th^ gestational week and died shortly after. No associations were found between groups regarding the prevalence of congenital malformations, neonatal jaundice, hypoglycemia, LGA diagnosis and ten-minute Apgar score. To note, no difference was observed regarding the length of neonatal hospital stay after birth between Index and Comparison groups; the latter did not differ even after studying a subgroup of solely non-healthy children (data not shown).

**Table 2:** Neonatal outcomes of infants conceived through oocyte donation (Index group) or conceived spontaneously (Comparison group A)

	Index group		Comparison group A			
	Median (IQR)	Range	Median (IQR)	Range		
Gestational length	40 (4)	28–42	40 (3)	28–43		
Head circumference, cm	35 (3)	25–38	35 (2)	25–40		
Birth Length, cm*	50 (3.5)	39–54	51 (3)	32–56		
Birth Length term infants, cm	50 (4)	45–54	51 (3)	42–56		
Birthweight, grams*	3238 (840)	1105–4910	3495 (693)	730–5800		
Birthweight term infants, grams	3380 (795)	2284–4910	3512 (668)	2200–5800		
	n	%	n	%	Unadjusted OR (95 % CI)	Adjusted^a^ OR (95 % CI)
Perinatal death (<7 days after birth)	1/76		0/149			
Congenital malformation^a^						
No	68/72	94.4	134/139	96.4	1.58	1.37
Yes	4/72	5.6	5/139	3.6	(0.41–6.06)	(0.23–8.14)
5 min Apgar s^a^						
≥7	70/75	93.3	148/149	99.3	10.57	7.01
<7	5/75	6.7	1/149	0.7	(1.21–92.20)	(0.40–123.43)
10 min Apgar s						
≥7	74/75	98.7	148/148	100.0	–	–
<7	1/75	1.3	0	0.0		
Asphyxia^a^						
No	70/76	92.1	145/149	97.3	3.11	2.49
Yes	6/76	7.9	4/149	2.7	(0.85–11.37)	(0.52–12.00)
Preterm delivery (<37w)^b^						
No	63/76	82.9	137/149	91.9	2.36	2.24
Yes	13/76	17.1	12/149	8.1	(1.02–5.45)	(0.87–5.81)
LGA^b^						
No	74/75	98.7	145/149	97.3	0.49	0.32
Yes	1/75	1.3	4/149	2.7	(0.05–4.46)	(0.03–3.46)
SGA^b^						
No	69/75	92.0	146/149	98.0	4.23	3.39
Yes	6/75	8.0	3/149	2.0	(1.03–17.42)	(0.59–19.69)
Low birthweight^a^						
<2500 gr	8/76	10.5	8/149	5.4	2.07	0.65
≥2500 gr	68/76	89.5	141/149	94.6	(0.75–5.76)	(0.10–4.30)
Jaundice^a^						
No	74/76	97.4	146/149	98.0	1.32	1.62
Yes	2/76	2.6	3/149	2.0	(0.22–8.05)	(0.23–11.68)
Hypoglycemia^a^						
No	73/76	96.1	145/149	97.3	1.49	1.74
Yes	3/76	3.9	4/149	2.7	(0.33–6.83)	(0.25–12.03)
Gender of the child^a^						
Boy	39/76	51.3	71/149	47.7	0.86	0.85
Girl	37/76	48.7	78/149	52.3	(0.50–1.50)	(0.45–1.60)
Infant Hospital stay, days^a^						
0–2	41/68	60.3	102/139	72.3	1.72	1.13
≥3	27/68	39.7	39/139	27.7	(0.94–3.17)	(0.56–2.31)

^a^Adjusted for maternal age when giving birth (< or ≥ 35 yrs), maternal BMI (< or ≥25 kg/m^2^), gestational age (continuous variable) and cesarean section (no/yes)

^b^Adjusted for maternal age when giving birth (< or ≥ 35 yrs), maternal BMI (< or ≥25 kg/m^2^) and cesarean section (no/yes)

* *p* < 0.05

**Table 3:** Neonatal outcomes of infants conceived through oocyte donation (Index group) or conceived through conventional IVF (Comparison group B)

	Index group		Comparison group B			
	Median (IQR)	Range	Median (IQR)	Range		
Gestational length	40 (4)	28–42	39 (2.3)	36–42		
Head circumference, cm	35 (3)	25–38	35 (3)	30–38		
Birth Length, cm	50 (3.5)	39–54	50 (3.3)	44–56		
Birth Length term infants, cm	50 (4)	45–54	50 (3)	44–56		
Birthweight, grams	3238 (840)	1105–4910	3545 (785)	1985–5420		
Birthweight term infants, grams	3380 (795)	2284–4910	3585 (743)	1985–5420		
	n	%	n	%	Unadjusted OR (95 % CI)	Adjusted OR (95 % CI)
Perinatal death (<7 days after birth)	1/76		0/63			
Congenital malformation^a^						
No	68/72	94.4	56/60	93.3	0.82	0.32
Yes	4/72	5.6	4/60	6.7	(0.20–3.44)	(0.05–2.05)
5 min Apgar s^a^						
≥7	70/75	93.3	62/63	98.4	4.43	1.24
<7	5/75	6.7	1/63	1.6	(0.50–38.94)	(0.11–14.18)
10 min Apgar s						
≥7	74/75	98.7	61/61	100.0	–	–
<7	1/75	1.3	0	0.0		
Asphyxia						
No	70/76	92.1	63/63	100.0	–	–
Yes	6	7.9	0	0.0		
Preterm delivery (<37w)^b^						
No	63/76	82.9	60/63	95.2	4.13	4.35
Yes	13/76	17.1	3/63	4.8	(1.12–15.21)	(1.08–17.52)
LGA^b^						
No	74/75	98.7	60/63	95.2	0.27	0.09
Yes	1/75	1.3	3/63	4.8	(0.03–2.67)	(0.01–1.06)
SGA^b^						
No	69/76	92.0	62/63	98.4	5.39	1.70
Yes	6/76	8.0	1/63	1.6	(0.63–46.04)	(0.16–17.72)
Low birthweight^a^						
<2500 gr	8/76	10.5	1/63	1.6	7.29	0.50
≥2500 gr	68/76	89.5	62/63	98.4	(0.89–59.99)	(0.02–13.81)
Jaundice^a^						
No	74/76	97.4	59/63	93.7	0.40	0.32
Yes	2/76	2.6	4/63	6.3	(0.07–2.25)	(0.04–2.44)
Hypoglycemia^a^						
No	73/76	96.1	60/63	95.2	0.82	0.44
Yes	3/76	3.9	3/63	4.8	(0.16–4.22)	(0.07–2.90)
Gender of the child						
Boy	39/76	51.3	31/63	49.2	0.92	0.70
Girl	37/76	48.7	32/63	50.8	(0.47–1.79)	(0.32–1.52)
Infant Hospital stay, days^a^						
0–2	41/68	60.3	40/58	69.0	1.46	0.94
≥3	27/68	39.7	18/58	31.0	(0.70–3.06)	(0.38–2.30)

^a^Adjusted for maternal age when giving birth (< or ≥ 35 yrs), maternal BMI at first antenatal visit (< or ≥25 kg/m^2^), gestational age (continuous variable) and cesarean section (no/yes)

^b^Adjusted for maternal age when giving birth (< or ≥ 35 yrs), maternal BMI at first antenatal visit (< or ≥25 kg/m^2^) and cesarean section (no/yes)

More specifically, mean birthweight and length differed between Index and Comparison group A as a whole [(3238 ± 840) *vs* (3495 ± 693), (*p* = 0.013) and (50 ± 3.5) *vs* (51 ± 3), (*p* = 0.011) respectively]; nevertheless no differences were noted among term infants (i.e. gestational length ≥37 weeks at birth) in these groups [(3380 ± 795) *vs* (3512 ± 668), (*p* = 0.126) and (50 ± 4) *vs* (51 ± 3), (*p* = 0.056) respectively] (Table 2). However when the effect estimate was evaluated more thoroughly in a logistic regression model, the effect size was found to be subtle (i.e. OR close to one) and disappeared after adjusting for gestational length (data not shown). Additionally, the Index group had a higher incidence of SGA diagnosis (8 % *vs* 2 %, *p* = 0.064) and prematurity (17.1 % *vs* 8.1 %, *p* = 0.041) in relation to Comparison group A. Moreover, neonates conceived after oocyte donation had more frequently five-minute Apgar score (AS) below 7 compared to spontaneously conceived infants (6.7 % *vs* 0.7 %, *p* = 0.017), a portion of which were also born preterm (4 out of 5 neonates conceived through OD). However, the risk for the above conditions did not remain statistically significant after adjustment for the covariates named previously (i.e. maternal age, maternal BMI, gestational age, delivery by cesarean section) (Table 2).

Regarding infants conceived through oocyte donation or autologous IVF, no differences were noted with respect to head circumference, birthweight and length, independently if infants were born at term or preterm (*p* > 0.05) (Table 3). On the contrary, neonatal asphyxia (7.9 % *vs* 0 %, *p* = 0.032), low birthweight (<2500 gr) (10.5 % *vs* 1.6 %, *p* = 0.040) and prematurity (17.1 % *vs* 4.8 %, *p* = 0.031) were more frequently diagnosed among infants conceived through oocyte donation compared to Comparison group B; the above risks however did not differ after adjustment.

Finally, we compared the commonest neonatal outcomes after taking into account the medical indication of oocyte donation treatment. Our findings suggest that neonates born to women receiving treatment based on “other indication” have lower mean birthweight and length, as well as higher rate of SGA diagnosis, compared to neonates of women with diminished ovarian reserve or fertile women (Table 4). However no statistical difference is noted regarding birthweight and length when women with Turner syndrome are excluded from the “other indication” subgroup (data not shown). Five minute Apgar score below 7 (AS < 7 at 5 min) occurred more often on the “Diminished Ovarian Reserve” subgroup compared to “other indication of treatment” subgroup or Comparison group A (8.3 % *vs* 5.9 % *vs* 0.7 % respectively); a proportion of the latter can be associated to prematurity (i.e. preterm/total neonates with AS < 7 at 5 min: 2/3(66.7 %), 2/2(100 %) and 0/1(0 %) in each group respectively) (data not shown).

**Table 4:** Neonatal outcomes studied by indication of oocyte donation treatment compared to spontaneously conceived infants (Comparison group A)

	Comparison group A(CgA)		“Diminished ovarian reserve” subgroup (DOR)		”OD other” subgroup (ODo)		*p*-value CgA vs DOR	*p*-value Cga vs ODo
	n	(%)	n	(%)	n	(%)		
Congenital malformation	5/139	3.6 %	3/35	8.6 %	1/32	3.1 %	0.202	1.000
1 min Apgar s <7	9/149	6 %	5/36	13.9 %	4/34	11.8 %	0.110	0.266
5 min Apgar s <7	1/149	0.7 %	3/36	8.3 %	2/34	5.9 %	0.024	0.089
10 min Apgar s <7	0/148	–	0/36	–	1/34	2.9 %	–	0.187
Preterm delivery (<37w)	12/149	8.1 %	6/37	16.2 %	6/34	17.6 %	0.133	0.090
SGA diagnosis	3/149	2 %	1/36	2.8 %	5/34	14.7 %	0.583	0.006
	Median (IQR)	Range	Median (IQR)	Range	Median (IQR)	Range		
Head circumference	35 (2)	25–40	35 (2.75)	30–38	34.5 (4)	27–38	0.792	0.107
Birthweight	3472.5 (704)	730–5800	3550 (913)	2005–4910	3080 (801)	1105–4670	0.458	0.004
Birthweight term infants	3512 (668)	2200–5800	3622.5 (931.3)	2720–4910	3270 (686)	2284–4670	0.898	0.034
Birth Length	51 (3)	32–56	50 (5)	45–54	49 (3.25)	39–54	0.662	0.001
Birth length term infants	51 (3)	42–56	51 (4.25)	45–54	50 (2.75)	45–54	0.832	0.011

“Diminished ovarian reserve” subgroup includes women with Premature Ovarian Insufficiency (POI) or those who are poor responders

”OD other” subgroup includes all other indications (i.e. women with Turner syndrome, after oophorectomy or chemotherapy, genetic reasons, multiple unsuccessful IVF, ”egg factor”)

## Discussion

Our data suggest that neonates conceived through oocyte donation are more frequently born preterm (<37 weeks) and have a lower mean birthweight and length compared to infants conceived spontaneously. The somatic measurements are nonetheless improved among term infants independently of mode of conception. The results that are of borderline statistical significance indicate a notable trend but possibly reflect the limited sample size. Although our findings conform to those of previous published studies [4–6], as well as with the latest and largest meta-analysis in the field [18], one can still reflect on the clinical significance of such subtle birthweight differences. It seems however safer to conclude that there is an increasing body of evidence pointing in the direction of a pragmatic negative association between oocyte donation-gestational length and fetal growth which may be considered much more important in the long term prognosis of these infants.

Notably, a higher prevalence of hypertensive disorders of pregnancy is observed among women conceiving after oocyte donation [7, 8]. It still remains unclear whether and at which degree hypertension or preeclampsia in relation to the mode of conception might act synergistically affecting gestational length or leading to growth restriction due to utero-placental insufficiency.

Contrary to other studies [2, 4], a five-minute Apgar score below 7 was more frequent among infants conceived through oocyte donation; the effect, however, disappears when gestational age is taken into account, possibly reflecting the effect of prematurity. No differences were noted regarding the presence of congenital malformations in our study; one should however be cautious in the interpretation of these results since that might reflect limited statistical power.

Further analyses within the oocyte donation group revealed that birthweight and length is lower after pregnancies with treatment indications other than diminished ovarian reserve. Our finding, which comes in contrast to the study by Keegan et al. [26], can possibly be attributed to the high proportion of women with Turner syndrome in our sample, since no neonatal somatometric differences are noted after excluding those women. However, women with Turner syndrome constitute an important part of the oocyte recipient population; we chose as a consequence not to exclude them from the study population and the analysis presented. To note, neonatal outcomes similar to those in the Turner subgroup in our study were observed in the Turner syndrome study by Hagman et al. carried out in Scandinavia [25]. Finally, in contrast to Anttila et al. [14] and Cobo et al. [15], the duration of hospital stay in a newborn surveillance unit did not differ between Index and Comparison groups.

Although the exact underlying pathophysiological mechanisms remain obscure, OD pregnancies show a greater degree of antigenic dissimilarity i.e. HLA mismatch in peripheral blood compared to IVF with autologous oocytes or spontaneously conceived pregnancies [29, 30]. Placentas from OD pregnancies, examined histologically and immunohistochemically, exhibited increased diffuse chronic deciduitis with dense fibrinoid deposition in the basal plate of the placenta, as well as increased infiltration by mononuclear cells compared to non-donor IVF pregnancies [31]. The corresponding pattern of immune mediated placental pathology is considered a unique sign of donated oocyte conception and is postulated to be representative of a type of ”host-versus-graft reaction” [4].

The principal strength of this report lies in its research design, being a national-level study. Since it was not carried out at a single setting for ART, the results are not reflective of specific treatment protocols or embryology laboratory techniques. Regarding reproductive health, the public health insurance program in Sweden covers all gamete donation treatment costs, providing the opportunity for citizens to equally benefit, independent of their financial ability. Moreover, the maternal health care system is organized within a well-developed and easily available primary care sector with standardized and free of charge antenatal care carried out mainly by community midwives with referral for obstetric assessment by physicians when potential complications are detected [32]. Thus, the contrasting outcomes cannot be solely attributed to the different level of obstetric or neonatal care provided to the three study groups. Furthermore, previously reported unfavorable findings have been questioned due to the presence of multiple confounding factors (i.e. maternal comorbidities, advanced maternal age, multiple gestations) [13, 18]. Luckily, the latter does not apply for the nationwide oocyte donation program in Sweden. The participating University clinics that make the eligibility evaluation of the recipients, seem to be in agreement regarding the importance of age and weight restrictions (age < 40 years and BMI < 35 kg/m^2^), as well as good health status of the oocyte recipients [33]. It is therefore important to note that the neonatal outcomes in our index population were observed in spite of the eligibility criteria adopted by healthcare practitioners in Sweden, introducing oocyte donation as an independent risk factor.

The limitations of the study include the limited sample size and low statistical power as seen by the wide confidence intervals; thus, further studies in similar settings are needed to confirm our findings. On the other side, there is only one similar study which comprised Finnish participants, but it was smaller, dates back to 1998, and uses only IVF pregnancies as control group [14]. Based on the fact that most studies included in a recent meta-analysis come from the United States [18], we feel that the information presented herein, originating from a country with a different health care system, might be a valuable input in the literature and can contribute in more robust conclusions in future review and meta-analytic approaches. Although we do not report a large enough series on infants conceived through oocyte donation treatment to permit statistically safe conclusions, our results conform to the latest international scientific data [18].

Additionally, a possible limitation is that the birthweight analysis in this study was not adjusted for cryopreservation (i.e. fresh *vs* cryopreserved embryos) or in vitro culture length, which are both believed to affect birthweight probably through epigenetic alterations. However, current data suggest that neither birthweight, nor preterm delivery among infants conceived after oocyte donation differ between fresh or frozen-thawed cycles [15, 34, 35] or between embryos transferred at different developmental stages [34]. Finally, although outcome variables in the Swedish national health registers are regarded as highly valid [21], the lack of more detailed information from participants’ hospital medical records remains a limitation.

## Conclusion

Infants conceived after oocyte donation have higher odds of prematurity and lower mean birthweight in this nation-wide study, in spite of the generally good health status and relatively young age of the Swedish oocyte recipients. Although the findings are of concern, their clinical relevance is still dubious. Couples receiving treatment with donated oocytes should nonetheless receive preconception counseling and individualized antenatal monitoring.

## Abbreviations

ART: Assisted reproductive technology
AS: Apgar score
CS: Caesarean section
IVF: In vitro fertilization
LGA: Large for gestational age
MBR: Medical birth register
OD: Oocyte donation
POI: Premature ovarian insufficiency
SGA: Small for gestational age.
